# Assessing Zoonotic Risks of *Blastocystis* Infection in Singapore

**DOI:** 10.3390/pathogens14080773

**Published:** 2025-08-05

**Authors:** Thet Tun Aung, Charlotte Kai Qi How, Jean-Marc Chavatte, Nazmi Bin Nazir, Edgar Macabe Pena, Bryan Ogden, Grace Rou’en Lim, Yasmina Arditi Paramastri, Lois Anne Zitzow, Hanrong Chen, Niranjan Nagarajan, Kevin Shyong Wei Tan, Benoit Malleret

**Affiliations:** 1 Department of Microbiology and Immunology, Immunology Translational Research Programme, Yong Loo Lin School of Medicine, National University of Singapore, Singapore 117545, Singapore; tta@nus.edu.sg (T.T.A.);; 2 Singapore Eye Research Institute, Singapore 169856, Singapore; 3 National Public Health Laboratory, National Centre for Infectious Diseases, Singapore 308442, Singapore; jean-marc_chavatte@cda.gov.sg; 4 SingHealth Experimental Medicine Centre and National Large Animal Research Facility, Singapore 169856, Singapore; edgar.macabe.pena@singhealth.com.sg (E.M.P.); bryan.ogden@singhealth.com.sg (B.O.); 5 Comparative Medicine, National University of Singapore, Singapore 119077, Singapore; grace_l@nus.edu.sg (G.R.L.); yasmina@nus.edu.sg (Y.A.P.); lois.zitzow@unibe.ch (L.A.Z.); 6 Genome Institute of Singapore, A*Star, Singapore 138672, Singapore; chen_hanrong@gis.a-star.edu.sg (H.C.); nagarajann@gis.a-star.edu.sg (N.N.); 7 Department of Microbiology and Immunology, Healthy Longevity Translational Research Programme, Yong Loo Lin School of Medicine, National University of Singapore, Singapore 117545, Singapore; mictank@nus.edu.sg

**Keywords:** zoonosis, *Blastocystis* spp., macaque

## Abstract

*Blastocystis* spp. is an enteric protist that is present worldwide. Despite being discovered a century ago, there is still much to be learned about its pathogenicity and transmission. Different subtypes (ST) of *Blastocystis* spp. have been identified in various hosts, including humans, birds, and insects, and there is potential for zoonotic transmission through contact between humans and animals. The prevalence of *Blastocystis* spp. in humans and macaques in Singapore was understudied, and the findings revealed a significant prevalence of the parasite, with rates of 90% and 100% observed in each respective *Macaca fascicularis* population 1 and 2, with main subtypes (ST1, ST2, ST3, and ST5). Using metagenomics, the different subtypes of *Blastocystis* spp. (comprising ST2, ST3, and ST17) were identified in a healthy Singaporean cohort. Additionally, seven incidental findings of *Blastocystis* spp. were discovered in human patients with other gut parasites, including two ST1, two ST2, two ST3, and one ST8. Several factors such as diet or reverse zoonotic transmission are suggested to play a role in *Blastocystis* sp. subtype distribution.

## 1. Introduction

*Blastocystis* Alexeieff, 1911 is an anaerobic eukaryotic protist that inhabits the gastrointestinal tract of various hosts, including humans [1], mammals [2,3], birds [4], and insects [5]. Its prevalence in humans varies globally, with rates ranging from 22–56% in European countries to 37–100% in Asian and American countries [6]. Symptoms of *Blastocystis* spp. infections typically include diarrhea and irritable bowel syndrome [7]. However, instances of asymptomatic individuals have been reported [8]. Conversely, studies have indicated a positive correlation between a healthy gut microbiome and *Blastocystis* spp. colonization [9]. Thus, the parasite’s classification as either a commensal or a pathogen remains uncertain, making its clinical significance unclear.

While *Blastocystis* spp. naming used to be species-specific, no significant differences between isolates from different host species have been discovered [10]. This homogeneity between sequences is unexpected when one considers the extensive human–human or animal–human transmission that occurs, with animals forming a large reservoir for infection in humans [11]. Today, 18S SSU rRNA sequences are crucial in separating *Blastocystis* spp. isolates into subtypes (STs) for epidemiological studies.

At least 22 STs have been identified globally [12], with each ST possessing at least 4% divergence in sequences from other STs. ST occurrence depends on the species of the host and the geographical distribution. ST1–ST9 and ST12 have been isolated from humans, with ST4 being most common [13]. In Southeast Asia, the predominant *Blastocystis* spp. ST detected in humans was ST3 (Supplementary Figure S1).

ST5 is typically found in cows or pigs, ST6 in avian species, and ST8 in nonhuman primates [14]. The identification of ST5–ST8 in both animals and humans strongly supports transmission via zoonosis. As *Blastocystis* spp. subtypes have cryptic host specificity, more studies are required to fully elucidate the zoonotic potential of this parasite. The dominant subtypes of *Blastocystis* spp. vary across the different hosts ST1, ST2, and ST3 (humans); ST10 and ST5 (cows); ST1, ST2, and ST3 (monkeys); ST5 (pigs); ST7 and ST6 (hens); and ST3 and ST1 (rodents) [14].

Like many other gut parasites, *Blastocystis* spp. is primarily transmitted through the fecal–oral route, either by consuming contaminated food or water or by direct contact with infected feces [15]. In addition to human-to-human transmission, there have been documented cases of zoonotic transmission, with the parasite being transmitted from animals to humans. This is particularly evident in settings like zoos or research facilities, where close contact with animal reservoirs can occur [16].

Urbanization places humans much closer to wild animal habitats than before, resulting in humans now having more contact with wildlife than in the past, increasing the potential for zoonotic transmission of diseases. This can be seen in Singapore, where the long-tailed macaque (*Macaca fascicularis* Raffles, 1821), the most common nonhuman primate species, enters households and schools in search of food [17]. Previous studies have suggested that macaques are natural *Blastocystis* spp. hosts [2,18]. With increased interaction between humans and animals, these macaques become potential animal reservoirs for zoonotic pathogens. Moreover, *Blastocystis* spp. have been reported to have high prevalence rates of up to 50% in both livestock and captive animals [19]. In research, captive macaques are used as biomedical models due to similar biological and physiological functions with humans. Consequently, more investigation into *Blastocystis* spp. prevalence and effect on macaques is required.

This study is divided into two main areas. First, the prevalence and ST distribution of *Blastocystis* spp. in three populations of *M. fascicularis* from Vietnam or wild-caught (housed in Singapore research facilities) were examined, and several factors such as diet or reverse zoonotic transmission were analyzed to determine if they played a role in *Blastocystis* sp. subtype distribution. Second, metagenomic analysis was conducted on a human cohort from Singapore to determine *Blastocystis* sp. ST distribution, prevalence, and any potential association between physical characteristics and disease status. Finally, the STs of patients diagnosed with other gut parasites with an incidental finding of *Blastocystis* spp. from patients were determined.

## 2. Materials and Methods

### 2.1. Animals

#### 2.1.1. Description of 3 Populations of *M. fascicularis*

Fecal samples from a total of 101 nonhuman primates were analyzed during the study. The NHPs were from different populations as described below.

a. Population 1

Although 42 *M. fascicularis* samples were received from the same captive-breeding primate facility in Vietnam, the macaques were housed in 2 different facilities as they were being used for various research purposes (Supplementary Figure S2). After a mandatory quarantine period (t = 0 days), fecal swabs were obtained from 42 *M. fascicularis* when they arrived at facility 1. Shortly after, 4 *M. fascicularis* were shipped to facility 2. At 48 days, 30 fecal swabs were obtained from the original batch of *M. fascicularis* at facility 1. These 30 macaques were then euthanized after completing an unrelated IACUC approved research protocol. Last, at 182 days, 8 fecal *M. fascicularis* samples from facility 1 and 4 from facility 2 were obtained, making up the last 12 samples (Supplementary Figure S3).

b. Population 2

After arrival in Singapore years ago (ranging from 2 to 8 years depending on individual macaques), the macaques underwent a compulsory quarantine period before moving to facility 3 and were isolated for their entire stay in Singapore. Nine *M. fascicularis* fecal samples were collected from facility 3 at 2–8 years after arrival in facility 3 (Supplementary Figure S4).

c. Population 3

An exploratory study of detecting *Blastocystis* spp. was conducted on 50 *M. fascicularis* from the research facility.

#### 2.1.2. DNA Extraction from Fecal Samples

Three *M. fascicularis* populations (herein labeled population 1, population 2, and population 3) were selected from 2 different research facilities in Singapore. Fecal swabs (BD) were obtained from population 1 (*n* = 42), population 2 (*n* = 9), and population 3 (*n* = 42).

QiaAmp Fast DNA Stool Mini Kit (Qiagen, Venlo, The Netherlands) was utilized to extract DNA from fecal samples. The concentration, 260/230, and 260/280 values were determined using a NanoDrop 2000 spectrophotometer (Thermo Fisher Scientific, Waltham, MA, USA).

#### 2.1.3. Real-Time Polymerase Chain Reaction (qPCR)

Amounts of 0.1 µL of F2 (5′-CCTACGGAAACCTTGTTACGACTTCA-3′) primer, 0.1 µL of PrimeTime Eco Probe and 0.1 µL of F5 (5′-GGTCCGGTGAACACTTTGGATTT-3′) primer, 5 µL of PrimeTime Gene Expression Master Mix (Integrated DNA Technologies (IDT), Coralville, IA, USA), 1 µL of DNA sample, and 3.7 µL of nuclease-free water (Cytiva, Marlborough, MA, USA) were added to 8-well PCR strips (Thermo Fisher Scientific). qPCR was run on an ABI 7500 Real-time PCR system (Thermo Fisher Scientific). The cycle was as follows: −50 °C for 2 min, 95 °C for 10 min, and 40 cycles of 95 °C for 15 s followed by 60 °C for 1 min. The cut-off Ct value was 30 for *Blastocystis* spp.-positive samples.

#### 2.1.4. Polymerase Chain Reaction (PCR) Amplification

Amounts of 1.25 µL of 10 µM RD5 and BhRDr primers (Integrated DNA Technologies), 12.5 µL of HotStarTaq Master Mix (Qiagen), 1 µL of DNA sample, and 9 µL of nuclease-free water (Fisher Scientific) were added into 8-well PCR strips (Thermo Fisher Scientific). The samples were cycled through 95 °C for 15 min (for heat activation), then 30 cycles at 94 °C for 30 s, 60 °C for 30 s, and 72 °C for 1 min before 72 °C for 10 min.

#### 2.1.5. Gel Electrophoresis of PCR Products

An amount of 0.5 g of agarose powder (1st Base) was dissolved in 50 mL of 1× TAE buffer (Vivantis, Hyderabad, India) before 5 µL of SYBR Safe (Invitrogen, Thermo Fisher Scientific) was added to the mixture. The 1% agarose solution was poured into a gel tray with a 15-well comb and allowed to cool until solidified. An amount of 5 µL of GeneRuler 1 kb DNA Ladder (Thermo Scientific Scientific) was added to one lane for reference. Amounts of 5 µL of the PCR products and 1 µL of 6× New England BioLabs Gel Loading Dye (Ipswich, MA, USA) were mixed before being loaded into the wells. The gel was run at 100 V for 60 min. Agarose gel was imaged using a ChemiDoc XRS+ Gel Imaging System (BioRad, Hercules, CA, USA) for the visualization of *Blastocystis* spp.-positive bands at ~600 bp.

#### 2.1.6. Subtyping of *Blastocystis* spp.

qPCR positive *Blastocystis* spp. samples were sent to BioBasic Asia with forward (RD5) and reverse (BhRDr) primers for reference. The samples underwent Sanger sequencing. Sequences received in FASTA format were uploaded to PubMLST for subtype and allele identification.

For reference *Blastocystis* spp. sequence data used for phylogenetic analysis, at least 4 sequences of 18S SSU rRNA *Blastocystis* spp. isolates for ST1-ST8 (except ST6) were selected from humans, livestock, and nonhuman primates (NHP) in NCBI GenBank across various geographic regions. Sequence alignment was through ClustalW on MEGA X (Gap Opening Penalty (15.00) and Gap Extension Penalty (6.66)). Each alignment was trimmed at the beginning and end to improve the similarity scores. Similarity scores were used to plot phylogenetic trees using the Neighbour-Joining method in MEGA X. The best tree with the highest log likelihood was shown in each figure with bootstrap proportions at the nodes (100 replicates). Nucleotide substitution types were used with a maximum composite likelihood model. Trees were drawn to scale, with branch lengths measured as the number of substitutions per site.

### 2.2. Human

#### 2.2.1. Metagenomic Datasets and Data Processing

One hundred nine stool metagenomic datasets from the multi-ethnic Singaporean population were obtained from the Singapore Platinum Metagenome Project [20]. Paired-end sequencing was performed on the Illumina HiSeq4K platform (San Diego, CA, USA), obtaining 18–190 million 150 bp reads per sample. Hybrid metagenomic assemblies were constructed from Illumina short reads and nanopore long reads using OPERA-MS [21].

#### 2.2.2. Detection of *Blastocystis* spp. STs from Metagenomes

The short reads were mapped against 10 *Blastocystis* spp. genomes obtained from NCBI using BWA mem v0.7.17 [22]. The matches were filtered for 99% identity over 90% of the read length and ensured that reads are properly paired. *Blastocystis* spp. genome assemblies were divided into 10-kilobase (kb) windows, and the number of windows with at least one match were counted. Sample–genome pairs where >10% of genomic windows have nonzero coverage were flagged as indicating detection in the metagenome.

Separately, the short reads were also mapped against *Blastocystis* spp. rRNA full-length sequences obtained from PubMLST using bwa mem v0.7.17. The matches were filtered for 99% identity over 80% of the read length. Sample–gene pairs with mean BWA mapping quality > 5 were recorded. *Blastocystis* spp. 18S rRNA sequences were compared using BLAST+ 2.12.0 against metagenomic assemblies with an E-value threshold of 1 × 10^−50^ and >95% identity. The aligned contigs were extracted and utilized for phylogenetic analysis.

#### 2.2.3. Statistical Analysis

Measurements from a list of characteristics from each of the 109 samples were measured and recorded [23]. Characteristics from the 109 humans were analyzed across 2 groups—*Blastocystis* spp.-positive and *Blastocystis* spp.-negative for any significant associations. Statistical tests were run on GraphPad PRISM 9.3.1 (https://www.graphpad.com/features, accessed on 17 July 2025). Sex was tested for statistical significance using Fisher’s exact test (to compare between 2 different groups), while ethnicity was tested using Pearson’s Chi-square test (to compare the difference for more than 2 different groups). For the rest of the characteristics, each group (*Blastocystis* spp.-positive and *Blastocystis* spp.-negative) was first tested for normality using the Shapiro–Wilk test to determine if the populations are normal across each characteristic. Subsequently, either an unpaired parametric *t*-test (if normal distribution) or an unpaired nonparametric Mann–Whitney *t*-test (if not normal distribution) was carried out to determine if the *p*-values were significant. The difference was considered statistically significant if the *p*-value was less than 0.05.

#### 2.2.4. *Blastocystis* spp. Identification in Singapore Patients

Stool samples with morphological observations on fecal smears or positive detection by BioFire^®^ FilmArray^®^ GI Panel (BioFire Diagnostics, Salt Lake City, UT, USA) of enteritic Protozoa such as *Cyclospora* Schneider, 1881, *Cryptosporidium* Tyzzer, 1907, *Entamoeba* Casagrandi & Barbagallo, 1897, *Enterocytozoon bieneusi* Desportes, Lecharpentier, Galian, Bernard, Cochand-Priollet, Lavergne, Ravisse & Modigliani, 1985, or *Giardia* Künstler, 1882 were further tested in the Singapore National Public Health Laboratory (NPHL), by both microscopy and molecular methods. The presence of a suspected or detected primary parasite was confirmed and investigated for other enteritic parasites including *Blastocystis* spp. with subtyping as described in [24]; the ST was further confirmed by comparison with PubMLST.

## 3. Results

### 3.1. Subtyping and Phylogenetic Analysis of Blastocystis spp. in Population 1

After DNA extraction and analysis via qPCR, the *Blastocystis* spp. prevalence was determined. At 0 days, there was a *Blastocystis* spp. positive rate of 90.47% (38 out of 42). At 48 days, there was a *Blastocystis* spp. positive rate of 93.33% (28 out of 30). At 182 days, there was a *Blastocystis* spp. positive rate of 41.67% (5 out of 12).

At 0 days, the majority of *Blastocystis* spp.-positive samples were ST3 (42.1%), followed by ST1 (31.6%), ST2 (21.1%), ST5 (2.6%), and ST8 (2.6%). At 48 days, the majority of the samples were ST5 (78.5%), followed by ST3 (14.3%), then ST1 (3.6%) and ST2 (3.6%). Last, at 182 days, the majority of the samples were ST1 (80.0%) (Table 1).

**Figure 1:**
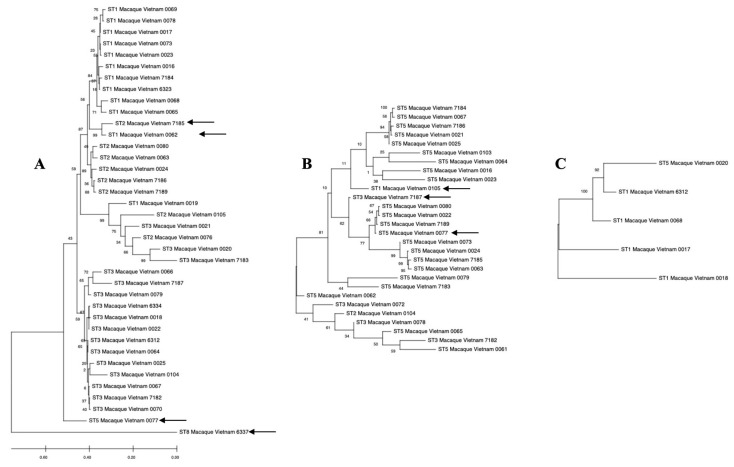
Phylogenetic relationships of the 18S small subunit (SSU) rRNA gene of *Blastocystis* spp. sequences inferred by Neighbour-Joining tree based on the 18S rRNA gene. The numbers on the branches are percentage bootstrap values of 100 replicates. Evolutionary distances between sequences were computed using the maximum composite likelihood method, and the horizontal branch is proportional to the estimated number of substitutions. (**A**) Phylogenetic tree from *Blastocystis* spp. sequences at 0 days. (**B**) Phylogenetic tree from *Blastocystis* spp. sequences at 48 days. (**C**) Phylogenetic tree from *Blastocystis* spp. sequences at t = 182 days. The black arrows indicate the samples that were either branched off or interspersed from their respective ST groups.

From the phylogenetic tree at 0 days (Figure 1A), ST1, ST2, and ST3 sequences from the *M. fascicularis* were mostly clustered together with relatively high bootstrap values. These three subtypes formed monophyletic clades. ST2 (sample 7185; indicated with a black arrow) was interspersed among a ST1 cluster. ST5 (sample 0077; indicated with a black arrow) branched off from the majority of the samples, along with ST8 (sample 6337; indicated with a black arrow). In the phylogenetic tree from 48 days (Figure 1B), ST5 sequences were mostly clustered together, with ST1 (sample 0105; indicated by a black arrow) and ST3 (Sample 7187; indicated by a black arrow) sequences interspersed. The horizontal branch lengths were longer, indicating that the ST5 sequences were evolutionarily distant from each other. Sample 0077 (indicated with a black arrow) was the only sample that retained ST5 subtype. Last, for the phylogenetic tree from the 182 days batch (Figure 1C), the five sequences are evolutionarily close to each other, with short branch lengths. There is high confidence in the plotted phylogenetic tree due to high bootstrap values.

### 3.2. Subtyping and Phylogenetic Analysis of Blastocystis spp. in Population 2

After DNA extraction and analysis via qPCR, *Blastocystis* spp. prevalence was determined. The *Blastocystis* spp.-positive rate was 100% (nine out of nine) (Table 2).

*Blastocystis* spp.-positive samples from facility 3 were evenly distributed across ST1 (33.3%), ST2 (44.4%), and ST3 (22.2%) (Table 2).

**Figure 2:**
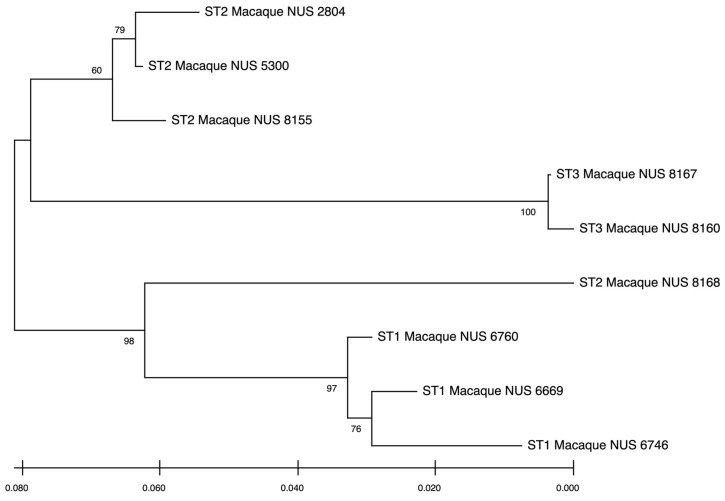
Phylogenetic relationships of one small subunit (SSU) rRNA gene of *Blastocystis* spp. sequences inferred by the Neighbour-Joining tree based on the 18S rRNA gene. The numbers on the branches are the percentage bootstrap values of 100 replicates. Evolutionary distances between sequences were computed using the maximum composite likelihood method, and the horizontal branch is proportional to the estimated number of substitutions. The phylogenetic tree is from *Blastocystis* spp. sequences from population 2. The black arrow indicates the branched off sample (sample 8168) from other ST2 samples.

For population 2 (Figure 2), ST1, ST2, and ST3 sequences were clustered together and were evolutionarily close to each other with short branch lengths. However, one of the ST2 sequences (sample 8168; indicated by a black arrow) branched off from the other ST2 sequences and was closer to the ST1 sequences.

### 3.3. Subtyping and Phylogenetic Analysis of Blastocystis spp. in Population 3

After DNA extraction and analysis via qPCR, the *Blastocystis* spp. prevalence was determined (42 out of 50 samples were *Blastocystis* spp. positive) (Table 3).

*Blastocystis* spp.-positive samples from population 3 were divided between ST1 (66.7%), ST2 (2.4%), ST3 (23.8%), ST5 (4.8%), and ST1 + ST3 (2.4%) (Table 3).

**Figure 3:**
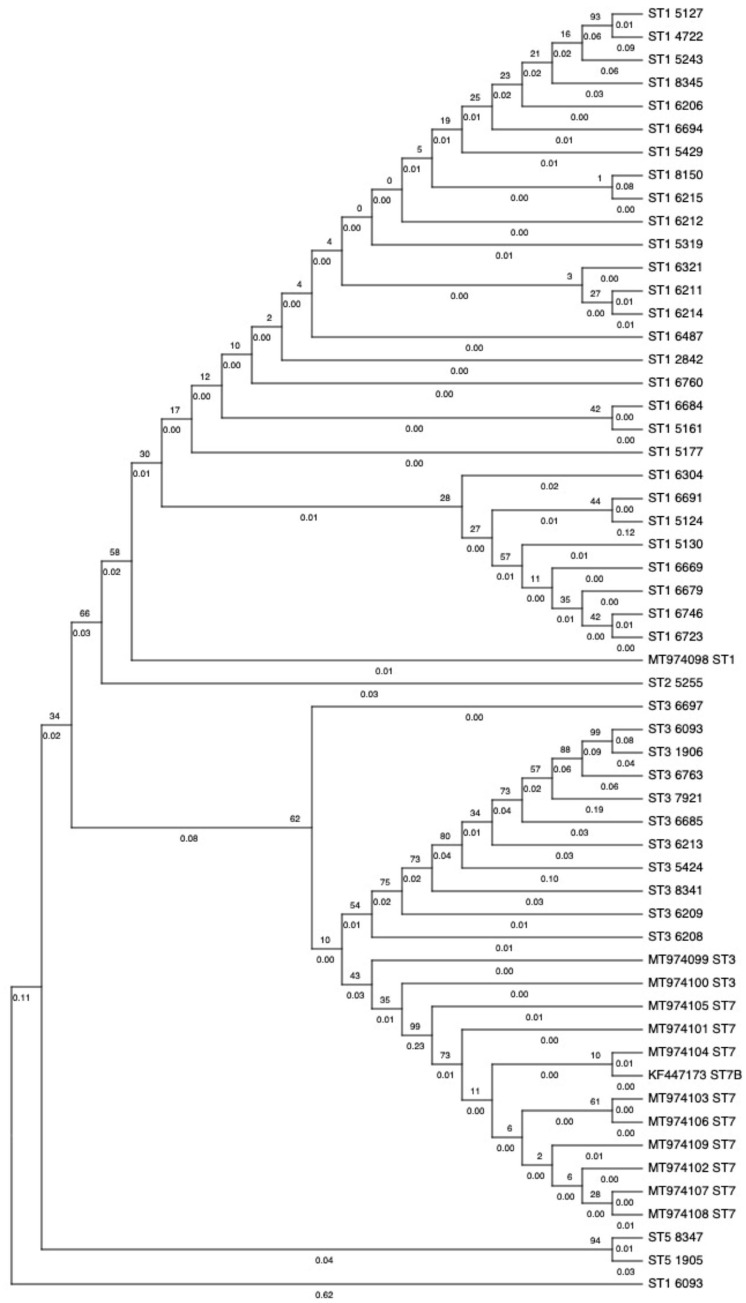
Subtype analysis and allele distribution of *Blastocystis* spp. Phylogenetic tree constructed using the maximum likelihood method, with a log likelihood of −9609.78. Bootstrap values are provided above the node, whereas the branch lengths are provided below the nodes. * represents the sample with co-infection (ST1 6093 and ST3 6093).

As seen from the phylogenetic tree (Figure 3), the majority of the branch lengths were 0.00–0.10, which indicated that the sequences were highly similar to each other. One hundred replicates were conducted with higher bootstrap values giving greater confidence in the observed branch. While some branches showed bootstrap values of more than 90, there were also some branches with values as low as 2. However, MEGA X computed the highest likelihood tree from the samples. With the exception of the co-infection sample, each subtype was clustered together and agreed well with subtyped sequences obtained from GenBank data.

Identification of *Blastocystis* spp. subtype distribution in two subgroups (wild caught and imported) from population 3 was performed. Hence, we reported that ST1 was the major subtype in both subgroups, followed by ST3 (Supplementary Figure S5).

### 3.4. Incidental Cases of Blastocystis spp.

Seven human cases with an incidental finding of *Blastocystis* spp. were identified from positive stool samples suspected or confirmed positive for other enteritic parasites such as *Cyclospora*, *Cryptosporidium*, *Entamoeba*, *Enterocytozoon*, or *Giardia*. They were discovered by microscopy on fecal smear, confirmed by molecular methods, and then subtyped. The different STs identified were as follows: 2 ST1, 2 ST2, 2 ST3, and 1 ST8 (Table 4).

### 3.5. Blastocystis spp. in Human Metagenomic Data

Prevalence of *Blastocystis* spp. and subtype distribution detected in metagenomic data.

Out of 109 samples taken from the multi-ethnic cohort in Singapore [20], there was a prevalence of 11.0% (12 out of 109) when compared against whole-genome sequences of *Blastocystis* spp. The majority of the samples were ST6 (58.3%; 7 out of 12), followed by ST3 (33.3%; 4 out of 12) and then ST2 (8.33%; 1 out of 12) (Table 5). However, 41.6% (5 out of 12) samples had a genomic window coverage of less than 80%. Additionally, there were a few samples with ≤30% match with ST6 sequences.

A low genomic window percentage coverage could be due to the presence of a different *Blastocystis* spp. ST whose genome was not in NCBI when we performed the analysis (Table 5). Hence, it was decided to match the reads against 18S SSU rRNA full-length sequences obtained from PubMLST.

**Figure 4:**
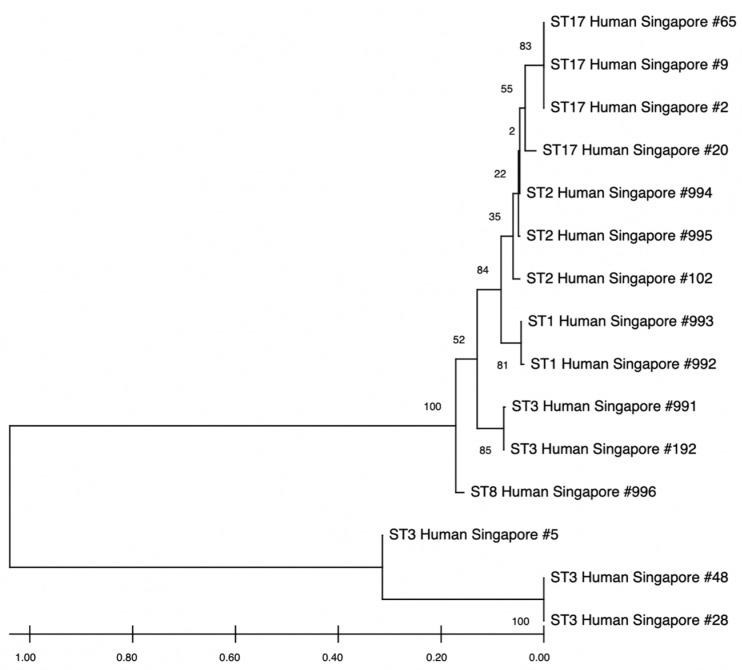
Phylogenetic tree of 18S small subunit (SSU) rRNA gene of *Blastocystis* spp. sequences pieced together from contigs from human gut samples from the Singapore Platinum Metagenome Project and incidental cases of *Blastocystis* spp. The tree was inferred by Neighbour-Joining based on the 18S rRNA gene. The numbers on the branches are percentage bootstrap values of 100 replicates. Evolutionary distances between sequences were computed using the maximum composite likelihood method, and the horizontal branch is proportional to the estimated number of substitutions.

There was a prevalence of 7.34% (8 out of 109) of *Blastocystis* spp. when comparing with 18S SSU rRNA full-length sequences from the same multi-ethnic cohort in Singapore [20] (Table 6). The majority of the samples were ST17 (50%), followed by ST3 (37.5%) and then ST2 (12.5%). All eight samples showed a high percentage identity with the query sequence (at least 95%) (Table 6). Apart from ST3 (sample ID-5), which branched off before the rest, the other sequences were quite clustered together according to subtype (Figure 4).

This is unusual as ST17 has mainly been identified in rodents (such as gundi, squirrel, and chinchilla) thus far [13,25,26,27], the probable natural hosts for ST17. Since the ST17 genome sequence is not available in NCBI, we could not corroborate the rRNA matches for these four samples of ST17-positive cases with the genomic analysis above. This dataset showing the identification of four samples that were positive for ST17 in human samples is unprecedented and could be an indicator of zoonotic transmission from rodents to human samples (Table 6).

### 3.6. Population Characteristics and Links to Blastocystis spp.

The dataset spanned diverse characteristics—from BMI and waist circumference to blood pressure and cholesterol levels (Table 7) [20]. Both *Blastocystis* spp.-positive and *Blastocystis* spp.-negative groups had similar distributions of sex and race (*p*-value > 0.05). The average and standard deviation across both populations were calculated, and the *p*-values were determined. The results were considered significant if the *p*-value was ≤0.05. From this table, the right apex neurothesiometer value was considered significant across both groups. Additionally, the total cholesterol had a low *p*-value of 0.072 compared with the rest of the metabolites.

## 4. Discussion

*Blastocystis* spp. is an enteric protist with widespread distribution throughout the world. In today’s global context, zoonotic diseases are gaining attention, especially with increased exposure to animal reservoirs. Hence, it becomes imperative that reservoirs of infection are identified from the community. In the local context of Singapore, one study determined the prevalence (3.3%; 9 out of 276) and subtype distribution (78% ST3 and 22% ST1) of *Blastocystis* spp. in patients from one of the hospitals in Singapore [28]. Additionally, another study determined the prevalence (10.1%; 25 out of 248) and subtypes (64% ST7; 16 out of 25) of *Blastocystis* spp. in patients with *Clostridioides difficile* (Hall & O’Toole, 1935) Lawson & Rainey, 2016 infection in Singapore [29]. However, no sampling of macaques or the healthy human population for *Blastocystis* spp. in Singapore had been conducted thus far.

In this study, five subtypes were identified across 51 *M. fascicularis* stool samples (population 1 and population 2)—ST1, ST2, ST3, ST5, and ST8. Additionally, many samples were not only of the same subtype but also the same allele. There was also a chronological comparison performed across six months for population 1, which enabled the investigation of how time affected the subtype distribution.

*Macaca fascicularis* is a nonhuman primate that is native to Southeast Asia and is one of the most popular choices as a nonhuman primate for research purposes due to suitability in reproductive and endocrine physiology [18,30]. There was a high prevalence of *Blastocystis* spp. present initially (90.47% (38 out of 42) (t = 0 days)) (Table 1). This result is comparable to other studies conducted in *M. fascicularis* in Asia [18].

High prevalence rates were expected as *M. fascicularis* are natural hosts of *Blastocystis* spp. and remain asymptomatic despite infection. Additionally, these macaques were held captive in an enclosed area, which could lead to an increase in *Blastocystis* spp. transmission. However, there was a notable drop in prevalence in the batch of 12 *M. fascicularis* at t = 182 days when the prevalence went from ≥90% to 41.67% (5 out of 12).

Although 47 *M. fascicularis* were housed individually, there were 4 that were socially housed at facility 2. The 51 *M. fascicularis* shared the same diet (i.e., commercial monkey chow and enrichment fruits from suppliers); however, enrichment (i.e., toys) was not shared across macaques.

To explain the loss in infections after a long time point (Table 1), one should first observe what was seen in the prospective study on *Blastocystis* spp. subtypes pre- and post-travel [31]. It reported that 27.6% changed subtype or lost infection after travelling and concluded that *Blastocystis* spp. carriage and transmission was highly dynamic—depending on the location of travel and various host factors. There are three other explanations for why 75% of the t = 182 days batch experienced a loss in infections. First, after a long period of time, there could be an alteration in the composition of the gut microbiota that causes disturbances in *Blastocystis* spp. growth conditions in the gut [32]. Second, it is possible that the natural *Blastocystis* spp. infection had run its course and been removed by the macaque immune system; however, currently, there is no consensus on how long *Blastocystis* spp. infection remains in the gut. Last, the socially housed macaques were younger in age, and all four of these macaques either lost the infection or did not acquire one due to their young age [33].

Sample 0077 was the only sample that was ST5 in population 1 (t = 0 days). Additionally, it maintained its ST5 subtype in population 1 (t = 48 days), which could have indicated a potential point source for contamination, (genome sequence percentage identity of 87.65% and e-value of 1 × 10^−113^ between t = 0 and t = 48 days). The majority of the macaques that changed subtype were living in separate cages with no intermingling (including sample 0077), hence warranting a deeper look into other commonalities.

Our results showed that 80% (20 of 25) of macaques that changed ST were changed to ST5.

First, food could have promoted ST5 proliferation, and enrichment fruits obtained from the suppliers may have been obtained from the wet market, which present a source for contamination [34,35]. Additionally, not only were they of the same ST5 subtype, but they also had the same allele (i.e., 115), which indicates a possible common environmental source of infection. Second, there could have been a low sensitivity for the method of detection. In this study, the utilized barcoding method that was suggested by Scicluna et al. (2006) (forward primer-generic and amplifying other eukaryotes) was more suitable for screening, rather than in-depth analysis of subtypes [36]. In fact, one study found that mixed-subtype infections are more common than detected when comparing next-generation sequencing methods with the conventional PCR method [37]. Although there is one study that found that stable *Blastocystis* spp. colonization is subtype-independent, it is possible that ST5 could have been in low abundance at the t = 0 day time point, and afterward, it proliferated by t = 48 days to stable gut colonization and outgrew other subtypes present in the gut [9].

Third, ST5 is typically considered a livestock subtype due to its high prevalence in pigs [38]. There were other animals held at facilities 1 and 2, including pigs and horses, which were located at another end of the facility.

Fourth, human subtypes obtained (ST1, ST2, and ST3) from *M. fascicularis* could be indicative of reverse zoonosis. This was supported by the higher prevalence of *Blastocystis* spp. infections in zookeepers, as well as similarity in subtype sequences obtained from both the animals and the zookeepers [2,39]. However, this is also rather unlikely as in a research facility, there are workflows in place to reduce the transmission of zoonotic organisms, and personal protective equipment (PPE) is not likely to be breached. Last, as macaques are social animals, living in isolation for a prolonged period of time could have increased their stress levels and exacerbated the pathogenicity of ST5 *Blastocystis* spp. [40].

For the nine *M. fascicularis* samples held in facility 3, there were only three subtypes identified: ST1 (33.3% (three of nine)), ST2 (44.4% (four of nine)), and ST3 (22.2% (two of nine)). It is worth noting that these macaques have been living at the facility for 2 to 8 years, and this proximity could have led to evolutionary closeness, as evidenced by short branch lengths (Figure 3). ST1, ST2, and ST3 are also typically considered as human subtypes and atypical of macaques, which provides further evidence of reverse zoonosis from the caretakers. Although contracting *Blastocystis* spp. from the caretakers in Singapore is unlikely due to extensive PPE and protective procedures, the level of compliance with PPE procedures at the macaque vendor’s breeding facility prior to import is unknown, and it is conceivable that *Blastocystis* spp. was contracted from the caretakers in Vietnam instead. It is possible that in the future, the subtype distribution of *Blastocystis* spp. in population 1 will continue to evolve to look more like population 2 (extrapolation of population 1).

All 51 *M. fascicularis* in this study (population 1 and population 2) were bred from the same breeding facility in Vietnam. Although the expectation is that wild-caught macaques would have higher subtype and allele variability in *Blastocystis* spp., this was not seen in population 1 when compared with population 3 (Supplementary Figure S5). Compared with population 3, there was a similar number of subtypes detected in population 1 but also a larger number of alleles, indicating allelic variability. Additionally, there was a mix of imported (*n* = 25) and wild-caught (*n* = 17) animals in population 3 (*n* = 42). As seen from Supplementary Figure S5, a significant proportion of macaques that were imported from Vietnam were ST1, followed by ST3. Wild-caught macaques from Singapore (this study) also displayed the same trend but at lower proportions. However, this research has low power due to the small sample size. For future studies, a comparison between imported and wild-caught macaques can be performed to determine if wild-caught would have a more diversified distribution of *Blastocystis* spp.

The Singapore Platinum Metagenomics Project [20], a multi-ethnic cohort analysis of 109 fecal samples, was the first metagenomic analysis of *Blastocystis* spp. for Singapore. Initially, the Illumina Short Reads were matched against *Blastocystis* spp. genomes found on NCBI and produced a prevalence of 11.0% (12 out of 109). However, many of the sequence matches had low percentage identity (Table 5), and whole-genome sequences were not available for every subtype on NCBI.

The decision was made to match the reads against 18S SSU rRNA full-length sequences obtained from PubMLST. All of the matches had ≥95% similarity (Table 6). This provided a final prevalence of 7.33% (8 out of 109), which was comparable to other studies conducted in Singapore. We note, however, that the metagenomics approach was less sensitive than the qPCR-based detection method in macaques. Since only 10 genomes were used for the genomic analysis, subtypes not in the reference database may have been missed (and may have caused the low % window coverage for 8 of the 12 samples). *Blastocystis* spp. present at low abundance may also cause a low % window coverage and/or lack of mapping to 18S rRNA.

The incidental detection of *Blastocystis* spp. among human stool samples suspected and/or confirmed positive for other enteritic parasites highlighted the regular occurrence of mixed pathogens infection as previously reported [24,30]. It is worth noting that since *Blastocystis* spp. is not included within the commercial syndromic gastro-intestinal molecular testing panels, its detection relies solely on microscopy, likely leading to a widely underestimated prevalence.

Interestingly, the subtyping of the incidental human cases confirmed the presence of ST1, ST2, and ST3, which are three dominant subtypes in human populations that have also been reported in animals, such as NHPs and rodents [6,14,41]. In addition, ST8 was also identified. This ST has been found in animals such as NHPs, rodents, birds, and arthropods [6]; is rarely found in humans [6]; and has not previously been reported from Singapore. Altogether, these results and a recent global report of widespread presence of *Blastocystis* spp. in rodents [41] support the idea of zoonotic transmission. Given this, more testing, subtyping, and implementation of a comprehensive monitoring of *Blastocystis* spp. to try to identify infections routes and reservoirs should be conducted. This could also shed light on the still controversial pathogenicity of the different *Blastocystis* spp. STs.

Based on the data collected from the human cohort (Table 7), only the right apex neurothesiometer value was significant (*p*-value = 0.043) when comparing *Blastocystis* spp.-positive and *Blastocystis* spp.-negative groups. While numerous studies have reported that *Blastocystis* spp.-positive individuals tend to have a lower BMI [42], the difference in BMI between the two groups in this study was not statistically significant (*p*-value = 0.186). Notably, Mirjalali et al.’s study was conducted in Iran, whereas Beghini et al.’s data were primarily from European or American populations [9,42]. Asians generally have lower BMIs compared with other populations worldwide, which may have contributed to the unexpected trend observed in this study.

To gain a more comprehensive understanding of the potential pathogenic role of *Blastocystis* spp., there are several studies that could be performed in the future. Gut microbiome analysis of *Blastocystis* spp. positive and negative patients can be compared for the presence of healthy gut microbiota differences to investigate whether *Blastocystis* spp. are part of the healthy gut or pathogens. Regular checking (monthly) of *Blastocystis* spp. subtype distribution from *M. fascicularis* is encouraged to determine the effects of zoonotic transmission over time. Finally, environmental swabs of the macaque’s living area, food and water provided, cages, and fecal swabs from the caretakers would elucidate the contamination pathways or potential reverse zoonosis transmission.

## 5. Conclusions

This study employed a comprehensive approach to analyze *Blastocystis* spp., incorporating molecular techniques such as subtype and phylogenetic analyses. This approach facilitated the determination of the prevalence and subtype distribution of *Blastocystis* spp. in *M. fascicularis* populations, indicating evidence of zoonosis. Additionally, metagenomics was also employed to determine the prevalence of *Blastocystis* spp. in the Singaporean population, revealing potential risk factors. Moreover, the incidental findings of *Blastocystis* spp. in humans with other parasites suggest that inclusion of *Blastocystis* spp. comprehensive testing could help investigate the controversial pathogenic role of the parasites by different STs, identify the possible infection routes, and possibly break the transmission cycle. By elucidating the zoonotic transmission pathways and capabilities of *Blastocystis* spp., tailored strategies for screening and controlling transmission can be developed for local contexts including animal facilities, providing valuable insights for public health initiatives. Through these findings, the study helps to bridge gaps in current *Blastocystis* spp. research, particularly concerning epidemiology and pathogenicity.

## Figures and Tables

**Table 1:** *Blastocystis* spp.-positive subtype distribution across *Macaca fascicularis* population obtained from population 1.

	0 Days (*n* = 38)		48 Days (*n* = 28)		182 Days (*n* = 5)	
	Number	Percentage	Number	Percentage	Number	Percentage
ST1	12	31.6%	1	3.6%	4	80%
ST2	8	21.1%	1	3.6%	-	-
ST3	16	42.1%	4	14.3%	-	-
ST5	1	2.6%	22	78.6%	1	20.0%
ST8	1	2.6%	-	-	-	-

**Table 2:** Table describing the *Blastocystis* spp.-positive subtype distribution across the *Macaca fascicularis* population obtained from population 2 (*n* = 9).

	Number	Percentage
ST1	3	33.3%
ST2	4	44.4%
ST3	2	22.2%

**Table 3:** Table describing the *Blastocystis* spp.-positive subtype distribution across the *Macaca fascicularis* population obtained from population 3 (*n* = 42).

	Number	Percentage
ST1	28	66.7%
ST2	1	2.4%
ST3	10	23.8%
ST5	2	4.8%
ST1 + ST3	1	2.4%

**Table 4:** Incidental cases of *Blastocystis* spp. detected among stool samples positive for other enteritic parasites.

Sample Year	Provider	Primary Diagnosis * (M/FA)	Microscopy Results	Molecular Results	*Blastocystis* spp. ST	GenBank Acc. No.
2016 ^‡^	NUH	*Cyclospora cayetanensis* Ortega, Gilman & Sterling, 1984 (M)	*C. cayetanensis* *Entamoeba* sp. *Blastocystis* spp. *Cryptosporidium* sp.	*C. cayetanensis* *Entamoeba hartmanni* Prowazek, 1912 *Blastocystis* spp. *Cryptosporidium hominis*/*parvum*	ST3	KX618192
2018	MOH	*Giardia lamblia* Lambl, 1859 (FA)	Negative *Blastocystis* spp.	*G. lamblia* *Blastocystis* spp.	ST3	PV335991
2018	MOH	*Entamoeba histolytica* Schaudinn, 1903 (FA)	*E. histolytica* *Blastocystis* spp.	*E. histolytica* *Blastocystis* spp.	ST2	PV335994
2020	TTSH	*Paragonimus* sp. Braun, 1899? (M) *Blastocystis* spp.? (M)	Negative *Blastocystis* spp.	Not confirmed *Blastocystis* spp.	ST1	PV335992
2020	NUH	Microsporidia (M) *Cryptosporidium* sp. (M)	Microsporidia *Cryptosporidium* sp. *G. lamblia*	*E. bieneusi* *Cryptosporidium hominis*/*parvum* *G. lamblia* *Blastocystis* spp.	ST1	PV335993
2023	KKH	*Salmonella* Lignieres, 1900 (FA) *G. lamblia* (FA)	n.a. *G. lamblia* *Blastocystis* spp.	n.a. *G. lamblia* *Blastocystis* spp.	ST2	PV335995
2024	KKH	*Cryptosporidium* sp. (FA) *G. lamblia* (FA)	*Cryptosporidium* sp. Negative *Blastocystis* spp.	*Cryptosporidium* sp. Not confirmed *Blastocystis* spp.	ST8	PV335996

* Primary diagnosis provided by clinical laboratories with M for microscopy and FA for BioFire^®^ FilmArray^®^ GI Panel; ^‡^ Case previously reported in [24].

**Table 5:** Table describing the corresponding subtype that matched to each sample with *Blastocystis* spp. whole-genome reference sequences from NCBI.

Sample	GenBank Accession Number	Subtype	% of Windows with Reads Mapped
5	GCA_000963385	3	99.75
13	GCA_000963415	6	26.97
18	GCA_000963415	6	32.83
27	GCA_000963415	6	26.28
28	GCA_000963385	3	100
44	GCA_000963385	3	90.11
46	GCA_000963415	6	12.41
48	GCA_000963385	3	99.84
52	GCA_000963415	6	26.59
83	GCA_000963415	6	27.10
88	GCA_000963415	6	50.03
102	GCA_000963365	2	95.46

**Table 6:** Table describing the corresponding subtype that matched to each sample with *Blastocystis* spp. 18S SSU rRNA full-length sequences obtained from PubMLST.

Sample	Query Sequence ID (PubMLST)	Subtype	Percentage Identity (%)
2	18S_rRNA_full_length_160	17	95.60
5	18S_rRNA_full_length_34	3	99.45
9	18S_rRNA_full_length_160	17	95.37
20	18S_rRNA_full_length_160	17	96.92
28	18S_rRNA_full_length_34	3	99.94
48	18S_rRNA_full_length_34	3	99.89
65	18S_rRNA_full_length_160	17	95.37
102	18S_rRNA_full_length_9	2	98.02

**Table 7:** The average (±standard deviation) of each characteristic measured in *Blastocystis* spp.-positive and *Blastocystis* spp.-negative populations along with the corresponding *p*-value from various statistical tests.

	*Blastocystis* spp.-Positive	*Blastocystis* spp.-Negative	*p*-Value
Number	8 (7.33%)	101 (92.67%)	-
Sex			
Female	5 (62.5%)	60 (59.4%)	>0.999
Male	3 (37.5%)	41 (40.6%)	
Race			
Chinese	3 (37.5%)	50 (49.5%)	0.673
Indian	2 (25.0%)	27 (26.7%)	
Malay	3 (37.5%)	24 (23.8%)	
Characteristics			
Age	53.4 ± 6.3	52.7 ± 5.8	0.769
Height (cm)	162.06 ± 9.58	161.49 ± 8.25	0.853
Weight (kg)	61.14 ± 15.08	65.9 ± 13.4	0.355
BMI (kg/m^2^)	23.22 ± 5.17	25.2 ± 4.26	0.186
Waist circumference (cm)	77.45 ± 10.65	82.72 ± 10.62	0.180
Hip circumference (cm)	94.48 ± 9.34	98.62 ± 8.51	0.157
Systolic blood pressure (mmHg)	115.31 ± 21.39	124.49 ± 27.3	0.367
Diastolic blood pressure (mmHg)	71.88 ± 10.37	79.36 ± 22.01	0.278
Neurothesiometer (Mv)			
Right apex	8.38 ± 4.09	6.06 ± 3.21	0.043
Left apex	7.31 ± 2.84	6.19 ± 3.33	0.166
Right medial malleolus	8.81 ± 3.81	7.82 ± 3.78	0.391
Left medial malleolus	7.94 ± 2.27	7.63 ± 3.82	0.347
Metabolites			
Fasting blood glucose (mmol/L)	5.06 ± 0.58	5.19 ± 0.67	0.951
Serum creatinine	61.38 ± 20.04	63.78 ± 18.07	0.619
Total cholesterol (mmol/L)	5.21 ± 0.67	5.8 ± 0.97	0.072
Triglycerides (mmol/L)	0.91 ± 0.57	1.23 ± 0.87	0.217
HDL (mmol/L)	1.37 ± 0.38	1.38 ± 0.36	0.970
Ratio of total blood cholesterol: HDL	4.02 ± 0.97	4.46 ± 1.32	0.590
LDL (mmol/L)	3.41 ± 0.44	3.77 ± 0.84	0.236
Glycosylated hemoglobin (DCCT %)	5.55 ± 0.46	5.68 ± 0.43	0.518

## Data Availability

The data that support the findings of this study are available on reasonable request from the corresponding author. Genetic sequences obtained from the incidental cases are deposited in GenBank.

## Supplementary Materials

The following supporting information can be downloaded at: https://www.mdpi.com/article/10.3390/pathogens14080773/s1, Figure S1. Diagram depicting the subtype distribution of *Blastocystis* spp. infections in humans in Southeast Asia. Figure S2. Phylogenetic trees of 18S SSU rRNA *Blastocystis* spp. sequences. (A) Complete sequences between *Blastocystis* spp. subtypes 1–9 obtained from NCBI [5]. (B) Partial *Blastocystis* spp. sequences ranging from 986 to 1983 bp obtained from PubMLST. (C) Partial *Blastocystis* spp. sequences ranging from 337 to 600 bp obtained from PubMLST. Best trees from the Neighbour-Joining method are displayed with bootstrap proportions (BPs) at each branch node. The branch length is proportional to the number of substitutions. Figure S3. Schematic describing the distribution of *M. fascicularis* (population 1) obtained from Vietnam by location and time. Figure S4. Schematic of timeline of arrival of *M. fascicularis* from Vietnam of population 1 and when fecal swabs were collected. Orange circles indicate time points at which fecal swabs were collected and subsequently analyzed. Figure S5. Timeline depicting the arrival of *M. fascicularis* from Vietnam of population 2 and when fecal swabs were collected. Orange circles indicate time points at which fecal swabs were collected and subsequently analyzed. References [43,44,45,46,47,48,49,50,51,52,53,54,55,56,57,58,59] are cited in the Supplementary Materials.

## Abbreviations

The following abbreviations are used in this manuscript:

ST	Subtypes
*M. fascicularis*	*Macaca fascicularis*
18S SSU rRNA	18S small sub-unit ribosomal RNA
PCR	Polymerase chain reaction
qPCR	Real-time polymerase chain reaction
NPHL	Singapore National Public Health Laboratory
NCBI	National Center for Biotechnology Information
BMI	Body mass index
NHP	Nonhuman primates
