# Supplementary material for: Assessing Zoonotic Risks of Blastocystis Infection in Singapore
Source: Pathogens. 2025 Aug 5;14(8):773. doi: 10.3390/pathogens14080773 (PMC12389398; doi:10.3390/pathogens14080773)
Supplement: Supplementary file 1 [file pathogens-14-00773-s001.zip › pathogens-3714013-supplementary.pdf]

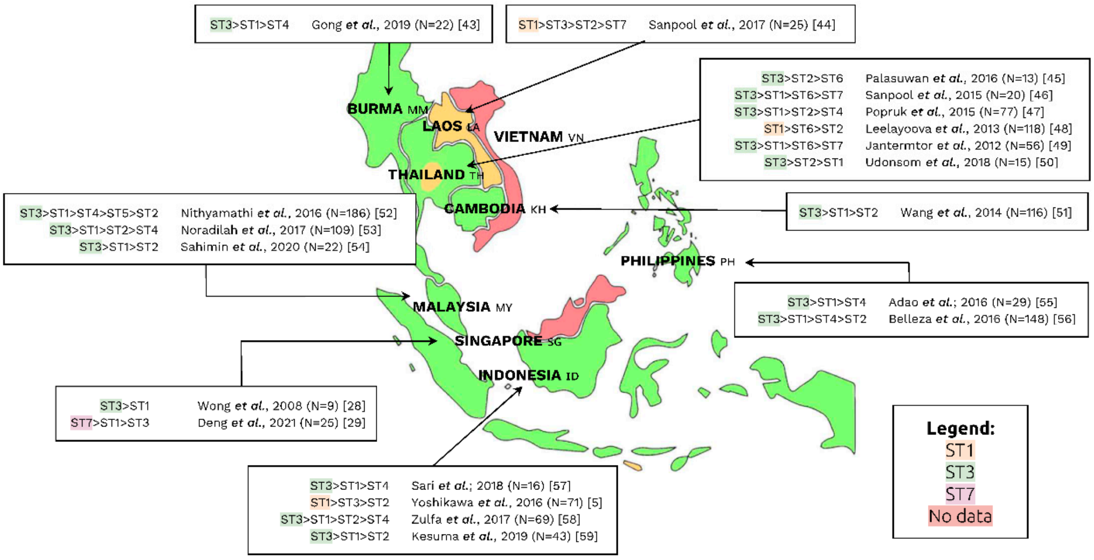

Supplementary Figure S1. Diagram depicting the subtype distribution of *Blastocystis spp.* infections in humans in South East Asia.

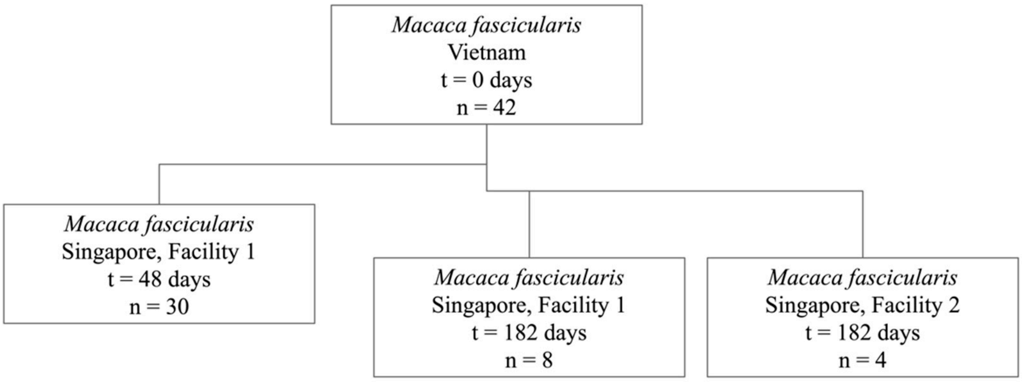

Supplementary Figure S2. Schematic describing the distribution of *M. fascicularis* (Population 1) obtained from Vietnam by location and time.

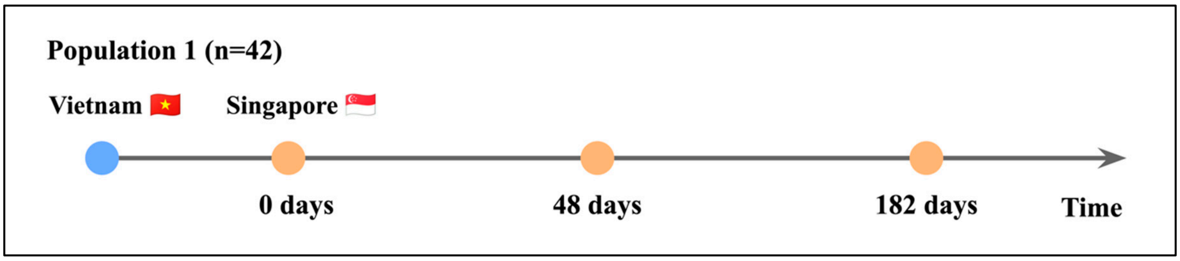

Supplementary Figure S3. Schematic of timeline of arrival of *M. fascicularis* from Vietnam of Population 1 and when fecal swabs were collected. Orange circles indicate timepoints which fecal swabs were collected and subsequently analysed.

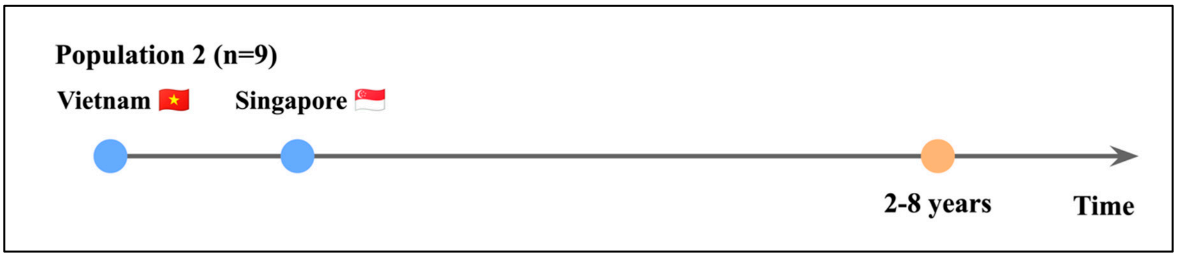

Supplementary Figure S4. Timeline depicting arrival of *M. fascicularis* from Vietnam of Population 2 and when fecal swabs were collected. Orange circles indicate timepoints which fecal swabs were collected and subsequently analysed.

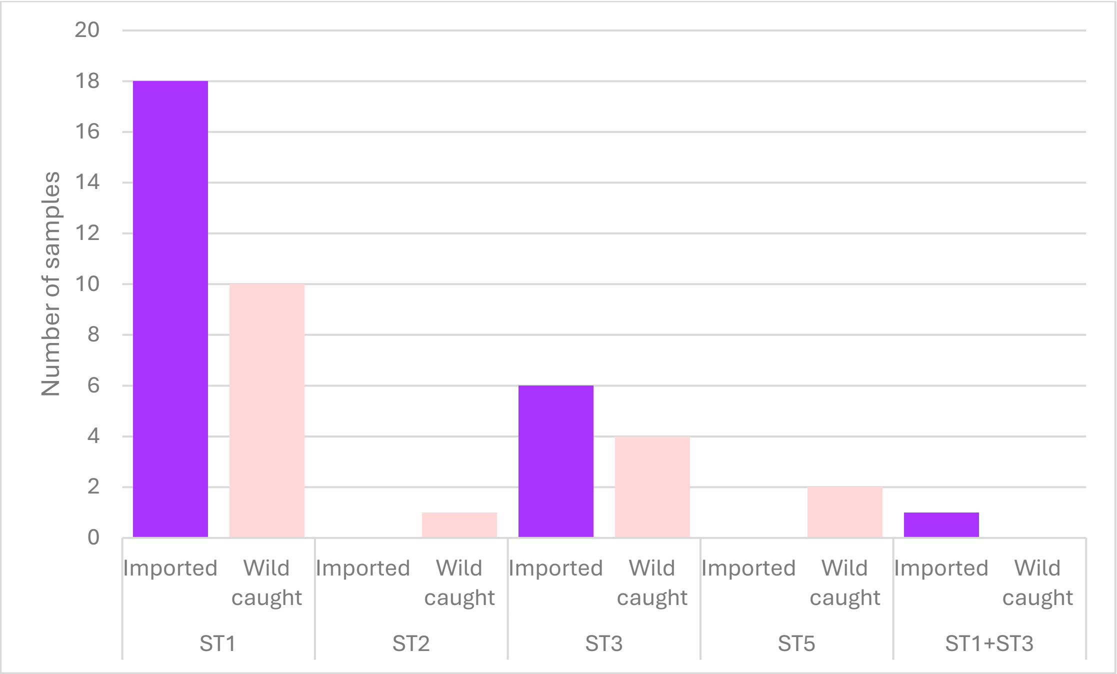

Supplementary Figure S5. Subtype distribution of *Blastocystis spp.* across imported and Singapore wild-caught *M. fascicularis* (n=42).
